# Utility of copeptin and standard inflammatory markers in the diagnostics of upper and lower urinary tract infections

**DOI:** 10.1186/s12894-015-0061-2

**Published:** 2015-07-08

**Authors:** Anna Masajtis-Zagajewska, Ilona Kurnatowska, Malgorzata Wajdlich, Michal Nowicki

**Affiliations:** Department of Nephrology, Hypertension and Kidney Transplantation, University Hospital and Education Centre of the Medical University of Lodz, Pomorska 251, 92-213 Lodz, Poland

**Keywords:** Copeptin, Urinary tract infection, Biomarker

## Abstract

**Background:**

A new serum marker of inflammation copeptin (CPP) a stable C-terminal pro-vasopressin was assessed along with conventional markers such as C-reactive protein (CRP), procalcitonin (PCT) and IL-6 to discriminate between lower and upper bacterial urinary tract infections (UTI).

**Methods:**

Study population comprised 45 patients including 13 with lower UTI (L-UTI) and 32 with upper UTI (U-UTI) and 24 healthy controls. Serum markers, blood cultures and urine cultures were assessed before commencing antibiotic treatment and repeated 24, 48 h and 7 days thereafter. Receiver operating curves (ROC) were plotted to assess a diagnostic utility of different inflammatory markers.

**Results:**

Before antibiotic therapy all inflammatory markers including serum CPP (2821.1 ± 1072.4 pg/ml vs. 223.8 ± 109.3 pg/ml; p < 0.05) were higher in UTI than in controls. CPP was not different between L- and U-UTI (2253 ± 1323 pg/ml vs 3051 ± 1178 pg/ml; p = 0.70) despite significant differences in hsCRP (2.09 ± 1.7 mg/dl vs 127.3 ± 62.4 mg/dl; p < 0.001), PCT (0.05 ± 0 vs 5.02 ± 0.03 ng/ml p < 0.001) and IL-6 (22.5 ± 1.6 vs 84.8 ± 67 pg/ml p < 0.001). For U-UTI the areas under the ROC curves were 1.0 for both hsCRP and CPP, 0.94 for PCT and 0.7 for IL-6 and for L-UTI 0.571, 1, 0.505 and 0.73, respectively. After 7 days of treatment all markers decreased in parallel to clinical response.

**Conclusion:**

Although elevated serum copeptin may become a marker of UTI it seems to be inferior compared to traditional serum inflammation markers for differentiation of bacterial infections involving upper and lower urinary tract.

## Background

Bacterial urinary tract infection (UTI) is the most common infection across all age groups. Although the part of the urinary tract involved, i.e., low or upper UTI needs to be quickly established this is not always possible if based on clinical symptoms only. Typically, clinical symptoms of lower (L-UTI) are dysuria, frequent and difficult or painful urination. In most cases clinical symptoms of upper UTI (U-UTI) are dominated by fever and side pain [1]. In the latter an accurate diagnosis and early treatment is crucial due to a risk of urosepsis and long-term consequences including chronic kidney disease [2, 3]. The duration of therapy recommended for L-UTI is shorter compared to a complicated UTI that involves the kidneys [4]. Thus there is a need to quickly and reliably identify the biomarkers which could be used not only for diagnostic purposes but also to determine the severity and location of the infection inside the urinary tract.

Arginine vasopressin (AVP) is one of the key hormones of cardio-vascular homeostasis and is an important part of an endocrine stress response resulting in a release of adrenal steroids [5]. Despite its dominant role in cardio-vascular disease, the measurement and diagnostic application of vasopressin have never been found useful in clinical practice due to methodological problems caused by its short half-life, functional interactions with platelets in serum and small molecular size [6, 7].

Copeptin (CPP), is a 39-amino acid glycosylated peptide [8]. Vasopressin and copeptin are derived from the same precursor protein – pre-pro-vasopressin, composed of 164 amino acids and consisting of single proteins: vasopressin, neurophysin II and copeptin [9]. Thus, copeptin is a C-terminal part of pro-vasopressin (CT-pro-AVP) and is released along with vasopressin. In contrast to vasopressin, copeptin is stable in serum or plasma in room temperature and it is relatively easy to measure [10, 11]. Several recent studies have investigated the usefulness of copeptin as a diagnostic and prognostic biomarker in several clinical conditions including the lower respiratory tract infections [12], septic shock [13], and stroke [14, 15]. To the best of our knowledge no study has systematically evaluated serum copeptin levels in both L-UTI and U-UTI. The study was carried out in order to assess the utility of a potential new serum inflammation marker copeptin in diagnosis of UTI compared to other inflammatory markers and to discriminate between lower and upper UTI.

## Methods

### Patients

The subjects for this single-center observational, prospective study were recruited from the patients manifesting symptoms of UTI who were consecutively admitted to our hospital from May 2011 through September 2012 and diagnosed with acute pyelonephritis, as well as among patients with symptoms of L-UTI who were treated in our outpatient department. The reference group included healthy subjects without any clinical and laboratory symptoms of infection. In all subjects from that group blood was taken to confirm that blood count and C-reactive protein level were within normal range. Also the urinalysis was carried out to confirm an absence of leukocyturia.

The diagnosis of UTI in the subjects from the study group was established on the basis of typical clinical symptoms and at least one positive urine culture. The inclusion criteria included the presence of acute symptoms like fever, flank pain (U-UTI) or pelvic pain (L-UTI), dysuria, urinary frequency and costo-vertebral angle tenderness (U-UTI), presence of pyuria. The exclusion criteria included the eGFR <30 ml/min/1.73 m^3^, presence of any concomitant organ or systemic infection, any hospitalization or surgical procedure within last 3 months, history of renal transplant and any permanent complicating factors of urinary tract (including complete obstruction, suspected or confirmed prostatitis) which cannot be effectively treated during the therapy of the infection and ongoing or recent (within last 7 days) antibiotic therapy. The patients with negative urine bacterial cultures and male subjects with perineal or rectal pain and perineal tenderness during the physical examination were excluded.

The Ethics Committee of the Medical University of Lodz approved the study protocol. All the subjects provided a written informed consent.

### Biochemical parameters

The following biochemical parameters were obtained before commencing an antibiotic treatment and all measurements were repeated according to the fixed schedule after 24, 48 h and 7 days. They included complete blood count, C-reactive protein (hsCRP), procalcitonin (PCT), IL-6, serum sodium, potassium, creatinine and serum copeptin. Urine and blood cultures were assessed on admission and after seven days of antibiotic therapy.

Circulating levels of copeptin were measured using a commercial ELISA kit from USCN Life Science (Houston, USA). All other parameters were measured with standard laboratory automated methods.

### Statistical analysis

All results are expressed as means ± SD and as median +/− interquartile ratio (IQR). Data distribution was checked by Kolmogorov – Smirnov test. Within-group comparisons were performed using *t*-test or non-parametric Wilcoxon test. For categorical variables, chi-square test or Fisher exact test were used. The cutoff points of all markers to optimum performance for differentiating L-UTI and U-UTI were determined by plotting the Receiver Operating Characteristic (ROC) curves [16]. Statistical analysis was performed using Statistica for Windows (version 10PL, StatSoft, Tulsa, OK, USA).

## Results

No clinically relevant side effects prompting a change or withdrawal of antibiotic therapy were observed.

### Patients

The study group comprised 45 patients (31 female, 14 male, age 50 ± 20 years) including 13 with L-UTI (10 female, 3 male, age 61 ± 24 years) and 32 with U-UTI (21 female, 11 male, age 54 ± 11 years). From 49 patients that were initially screened 4 were later excluded because of negative urine cultures thus resulting in 45 patients taken into the final analysis. The reference group included 24 healthy subjects (17 female, 7 male, age mean 50 ± 20 years).

### Biochemical parameters

Table 1 shows the clinical and biochemical characteristics of the participants. Before the introduction of the antibiotic therapy in UTI patients serum inflammation markers such as: hsCRP (91.3 ± 86.5 mg/dl), PCT (3.7 ± 15.3 ng/ml) and IL-6 (66.8 ± 81.6 pg/ml) were all significantly higher than in healthy patients (3.5 ± 2.3 mg/dl, 0.06 ± 0.02 ng/ml and 3.1 ± 1.6 pg/ml, respectively, p < 0.05 for all comparisons. Serum copeptin levels were also significantly higher in patients with UTI than in the controls (2821 ± 1072 vs. 223 ± 109 pg/ml; p < 0.05).

**Table 1:** Clinical and baseline biochemical characteristics of the study group with urinary tract infection (UTI) and control group

	UTI group	Control	P value
	(n = 45)	(n = 24)	
Sex			
Men	14 (31 %)	7 (29 %)	NS
Women	31 (69 %)	17 (71 %)	
Age (years)	56 ± 10	50 ± 20	NS
	[64 (39)]	[48.5 (33)]	
Creatinine (mg/dl)	1.3 ± 0.3	1.0 ± 0.3	NS
	[1.2 (0.4)]	[1.0 (0.3)]	
GFR (ml/min/1.73 m^3^)	73.1 ± 22	84.2 ± 26	NS
	[72.6 (39.5)]	[91.9 (41.6)]	
Leucocyte count (G/l)	12.8 ± 6.7	6.4 ± 2.1	<0.001
	[11.9 (6.9)]	[5.6 (3.3)]	
hsCRP (mg/L)	91.3 ± 86.5	3.5 ± 2.3	<0.001
	[98.2 (132.5)]	[3.3 (2.5)]	
Procalcitonin (ng/ml)	3.7 ± 15.3	0.06 ± 0.02	<0.001
	[0.12 (1.15)]	[0.05 (0)]	
IL −6 (pg/ml)	66.8 ± 81.6	3.1 ± 1.6	<0.001
	[41.6 (92.3)]	[2.8 (2)]	
Copeptin (pg/ml)	2821 ± 1072	224 ± 109	<0.001
	[874 (1527)]	[209 (141)]	

Results are expressed as mean ± SD [median (IQR)]

There were significant differences in serum hsCRP, PCT and IL-6 between L-UTI and U-UTI, but no difference in serum copeptin was observed (Table 2). Table 3 shows the comparison of biochemical parameters assessed at the time of the admission to the hospital emergency room in patients with the diagnosis of U-UTI. Statistical significant differences of these parameters between study and reference group were observed. Table 4 shows the differences of serum inflammatory markers between L-UTI and reference group. Only IL-6 and CPP differed significantly between the groups.

**Table 2:** Comparison of biochemical parameters assessed at hospital admission in patients with diagnosis of lower and upper urinary tract infection (UTI)

	Lower UTI	Upper UTI	P value
	(n = 13)	(n = 32)	
Leucocyte count (G/l)	8.6 ± 3.3	14.5 ± 5.9	0.005
	[7.4 (3.4)]	[12.7 (13.5)]	
hsCRP (mg/L)	2.9 ± 1.7	127.3 ± 62.4	<0.001
	[2.28 (2.81)]	[132.5 (124.8)]	
PCT (ng/ml)	0.05 ± 0	5.2 ± 0.03	<0.001
	[0.05 (0)]	[0.42 (0.9)]	
IL −6 (pg/ml)	22.5 ± 1.6	84.8 ± 67	<0.001
	[5.8 (41.9)]	[57.9 (64.3)]	
CPP (pg/ml)	2253 ± 1323	3051 ± 1178	Ns
	[1757 (1403)]	[2929 (2986)]	

Results are expressed as mean ± SD [median (IQR)]

**Table 3:** Comparison of biochemical parameters assessed at hospital admission in patients with diagnosis upper urinary tract infection (U-UTI) and control group

	Upper UTI	Control	P value
	(n = 32)	(n = 24)	
Leucocyte count (G/l)	14.5 ± 5.9	6.43 ± 2.1	<0.001
	[12.7 (13.5)]	[5.6 (3.3)]	
hsCRP (mg/L)	127.3 ± 62.4	3.5 ± 2.3	<0.001
	[132.5 (124.8)]	[3.3 (2.5)]	
PCT (ng/ml)	5.2 ± 0.03	0.06 ± 0.02	<0.001
	[0.42 (0.9)]	[0.05 (0)]	
IL −6 (pg/ml)	84.8 ± 67	3.1 ± 1.6	<0.001
	[57.9 (64.3)]	[2.8 (2)]	
CPP (pg/ml)	3051 ± 1178	224 ± 109	<0.001
	[2929 (2986)]	[209 (141)]	

Results are expressed as mean ± SD [median (IQR)]

**Table 4:** Comparison of biochemical parameters assessed at hospital admission in patients with diagnosis lower urinary tract infection (L-UTI) and control group

	Lower UTI	Control	P value
	(n = 13)	(n = 24)	
Leucocyte count (G/l)	8.6 ± 3.3	6.43 ± 2.1	NS
	[7.4 (3.4)]	[5.6 (3.3)]	
hsCRP (mg/L)	2.9 ± 1.7	3.5 ± 2.3	NS
	[2.28 (2.81)]	[3.3 (2.5)]	
PCT (ng/ml)	0.05 ± 0	0.06 ± 0.02	NS
	[0.05 (0)]	[0.05 (0)]	
IL −6 (pg/ml)	22.5 ± 1.6	3.1 ± 1.6	<0.001
	[5.8 (41.9)]	[2.8 (2)]	
CPP (pg/ml)	2253 ± 1323	224 ± 109	<0.001
	[1757 (1403)]	[209 (141)]	

Results are expressed as mean ± SD [median (IQR)]

On day 7 of antibiotic treatment, all serum markers including serum copeptin levels, with the exception of PCT, decreased significantly in patients with UTI along with the clinical response (Table 5). Copeptin levels did not completely return to normal range over the 7 days of treatment (2821 ± 1072 pg/ml on day 0 vs. 2003 ± 838 on day 7, p = 0.0001).

**Table 5:** Leucocyte count, IL-6, CRP, PCT and CPP levels during the treatment of UTI

	Day 0	Day 1	Day 2	Day 7	P value
Leucocyte count [G/l]	12.8 ± 6.7	10.5 ± 5.7	8.7 ± 3.9	6.6 ± 1.8	<0.001
	[11.9 (6.9)]	[8.9 (5.4)]	[7.3 (4.5)]	[6.7 (2.3)]	
IL-6 [pg/ml]	66.8 ± 81.6	70.6 ± 98.7	49.3 ± 68.4	34.4 ± 64.8	<0.001
	[41.6 (92.3)]	[22.9 (90.5)]	[12.6 (73.6)]	[8.7 (29.9)]	
hsCRP [mg/dl]	91.3 ± 86.5	77.6 ± 77.8	56.7 ± 72.7	13.5 ± 16.9	<0.001
	[98.2 (132.5)]	[57.6 (121.9)]	[25.29 (84.12)]	[7.4 (11.2)]	
PCT [ng/ml]	3.7 ± 15.3	1.8 ± 8.2	1.1 ± 4.6	0.4 ± 1.7	0.11
	[0.12 (1.15)]	[0.08 (0.5)]	[0.05 (0.2)]	[0.05 (0.03)]	
CPP [pg/ml]	2821 ± 1072	2309 ± 890	2290 ± 799	2003 ± 838	<0.001
	[2874 (1527)]	[2134 (934)]	[2140 (891)]	[1814 (1151)]	

Results are expressed as mean ± SD [median (IQR)]

**Table 6:** Areas the receiver operating characteristics (ROC) curves, 95 % Confidence Intervals (95 % CI) and cutoff points of optimum performance for differentiating upper from lower urinary tract

	Area under the ROC curve	95 % Cl	Cutoff point	Area under the ROC curve	95 % Cl	Cutoff point
	Upper urinary tract infection			Lower urinary tract infection		
hsCRP [mg/dl]	1	1.0 – 1.0	9.45	0.571	0.37 – 0.771	8.37
PCT [ng/ml]	0.94	0.845 – 0.993	0.07	0.505	0.185 – 0.575	0.15
IL-6 [pg/ml]	0.7	0.737 – 0.95	8.3	0.73	0.535 – 0.924	10.4
CPP [pg/ml]	1.0	1.0 – 1.0	1140	1.0	1.0 – 1.0	828

Receiver operating curves (ROC) were plotted to assess a diagnostic utility of inflammatory markers. As shown in Table 6 the biomarkers that best identified U-UTI were serum hsCRP and CPP followed by PCT and IL-6. In case of L-UTI the best performing biomarker was serum CPP followed by IL-6, hsCRP and PCT.

## Discussion

To date several studies have been conducted to confirm the hypothesis that copeptin, as a stress hormone, could also be a biomarker of several pathologic conditions including inflammation.

In our study a number of blood leucocytes, serum hsCRP, PCT, IL-6 and CPP significantly increased in patients with UTI compared to healthy subjects. However, trying to differentiate the patients with L-UTI from those with pyelonephritis serum copeptin unlike other inflammatory markers was not significantly different between these groups.

The value of the assessment of serum copeptin and other inflammatory markers in the diagnosis and differentiating between U- from L-UTI was also established by plotting the ROC curves. The area under the ROC curve of copeptin was equal in lower and upper urinary tract infection. It seems therefore that the measurement of serum copeptin seems to less clinically useful than the estimation of serum CRP and PCT to distinguish between L- and U-UTI.

Several earlier studies have investigated serum copeptin levels in different acute disorders. Serum copeptin was significantly higher in patients with lower respiratory system infection in comparison to healthy persons, with the highest level observed in patients with the community acquired pneumonia [12]. The patients with positive blood cultures had higher copeptin levels than patients with negative blood cultures. All our patients with UTI had positive urine cultures but negative blood cultures that did not allow the direct comparison of our results with the study of Muller et al. [12] that investigated the patients with more severe systemic infections.

Morgenthaler et al. assessed serum copeptin levels in patients with septic shock [13]. Increased copeptin was observed at time of hospital admission, including patients with different severity of sepsis compared with healthy control. Mean baseline serum copeptin was higher in patients who survived sepsis in comparison to those who died. Fluri at al. [14] performed the study comparing inflammatory markers and copeptin levels in patients suffering from stroke as early predictors for the development of post-stroke infection [14]. Serum procalcitonin, hsCRP, copeptin and leukocyte number were assessed as well as markers of pneumonia, UTI and other systemic infections. In that study mean levels of copeptin, CRP and number of leukocytes in blood in patients with UTI were similar to those with pneumonia and other infections with the exception of procalcitonin level that was lower in the former. In the patients with L-UTI in our study, increased levels of copeptin and IL-6 were observed but no other serum markers significantly differed from control group. That was not the case in patient with pyelonephritis, in whom serum levels of all markers were significantly higher compared to controls. Fluri et al. [14] concluded that copeptin, procalcitonin, CRP and number of blood leukocytes were good serum inflammatory markers in pneumonia and UTI however he did not divide their patients into L- and U-UTI. They also postulated that a combination of markers, including copeptin may be more helpful when making the decision about introducing a prophylactic antibiotic in patients with high risk of infection [14].

Copeptin levels were also measured in several studies performed in non-infectious diseases. Rechlin and Muller assessed the dynamic changes of copeptin levels in myocardial infarction [15]. In patients with myocardial infraction, copeptin levels increased within 4 h after the first symptoms, while troponin T levels still remained within normal range. Serum copeptin levels later decreased while troponin remained increased for the following several hours. The different kinetics of these markers could be used in the diagnosis of myocardial infraction. In our study we only assessed the changes of serum copeptin from the appearance of first symptoms of urinary tract infections to the day 7 of antibiotic treatment. Although high initial copeptin concentration was observed its decrease was slower compared to other inflammatory markers. Copeptin was still above normal at day 7 while the concentration of other inflammatory markers approached normal values at that time.

The potential biomarkers such as copeptin have to be always estimated in the context of a careful clinical assessment. Similar to many other biomarkers assessment of copeptin has certain limitations. There are possibly many factors which can lead to false – positive and false – negative serum copeptin results [16]. For example prednisone therapy increases copeptin concentration compared to healthy persons [17]. That was not the case in our patients since none was on steroids. Serum copeptin levels are also higher in patients with end-stage chronic kidney disease [18]. In our study however the patients with advanced kidney disease were excluded.

The use of new biochemical biomarkers that can simplify diagnostic and prognostic evaluation is not always economically feasible in particular since diagnostics and prognostics are customarily based on several different parameters. However, the usefulness of biomarkers is defined by the degree of their impact on clinical decision making and by adding subsequent information, apart from easily accessible data obtained from patients’ physical examination [19]. Although serum copeptin could be a clinically useful inflammation marker in case of UTI, our results show that it seems to be inferior compared to traditional serum inflammation markers for the differentiation of infections involving upper and lower part of the urinary tract.

The main limitation of our exploratory research performed in a single center was a relatively small group of patients.

## Conclusion

Although elevated serum copeptin may become a marker of UTI it seems to be inferior compared to traditional serum inflammation markers for differentiation of infections involving upper and lower urinary tract.

## Abbreviations

AVP: Arginine vasopressin
CPP: Copeptin
CT-proAVP: C-terminal part of provasopressin
hsCRP: C-reactive protein
L-UTI: Lower urinary tract infection
PCT: Procalcitonin
ROC: Receiver operating curves
UTI: Urinary tract infection
U-UTI: Upper urinary tract infection.
